# Tropical Oral Disease: Analysing Barriers, Burden, Nutrition, Economic Impact, and Inequalities

**DOI:** 10.3389/fnut.2021.729234

**Published:** 2021-11-22

**Authors:** Arvind Babu Rajendra Santosh, Thaon Jones

**Affiliations:** School of Dentistry, Faculty of Medical Sciences, The University of the West Indies, Kingston, Jamaica

**Keywords:** oral disease, dental caries, periodontitis, oral cancer, health care, nutrition, tropical, Caribbean

## Abstract

Traditionally, a healthy mouth is a good indicator of good general health. Poor oral hygiene reflects the health of the oral cavity and is a risk factor for overall health. Although oral diseases like dental decay and periodontitis are prevalent, awareness of oral diseases is still limited. Oral disorders include a wide range of diseases that may not be confined to the oral anatomical structures but may be manifestations of systemic diseases. Identification of the risk factors of dental and oral diseases, including socio-economic determinants, plays a major role in the type of oral health care, and in the promotion of dental health awareness. This article reviews oral diseases in the Caribbean and aims to raise awareness of this subject while suggesting a research agenda for the region.

## Introduction

Oral diseases can be broadly categorized into developmental, microbial, inflammatory, cystic, neoplastic, and oral manifestations of generalized or systemic diseases. The developmental pathologies may affect the soft and hard tissues of the oral cavity. Developmental malformations are the abnormalities that result from disturbances of growth and development (1, 2). The jaw bone, palate, dentition, and salivary glands may be involved in a number of disturbances that affect the shape and form of oral structures. Developmental alterations of the teeth and other structures lead to functional disturbances (3). However, greater levels of functional and aesthetic disturbances are associated with cleft lip and cleft palate. Some of the developmental disturbances are hereditary or familial due to mutations or genetic abnormalities, whereas other developmental disturbances are caused by local abnormalities or environmental influences (4). Studies on the frequency of developmental disorders of teeth show a wide range between 1.73 and 34.28% (5, 6). WHO report on the global burden of cleft-lip and/or palate data shows that an incidence of 3.74 per 1,000 live birth in Americans, 1:700 in Europeans,.82 and 4.04 per 1,000 live birth in Asians,.6 and 2.69 per 1,000 live birth in Caucasians, and 0.18 and 1.67 per 1,000 live births in Africans (7, 8). The highest incidence is found in Americans and the lowest in Africans, with an intermediate incidence in Caucasians.

The most frequent dental problem is dental caries which is microbial in origin. Dental caries result in the demineralization of inorganic constituents, whereas organic substances experience destructive damages. Infections in enamel and dentin tissue may further progress into the pulp and periapical tissues and cause serious dental problems such as pulpal and periapical inflammation (9). Periodontal diseases are infectious and inflammatory conditions primarily involve the periodontal tissues (10). These infections may spread into the jaw bone and soft tissues in favorable circumstances (11), for example, low tissue resistance, poor immune mechanism, or malnutrition. The presence of epithelial tissue within the bone marrow of the maxillae and mandible is one of the numerous dissimilarities between the jaws and other bones of the skeleton. The source of this epithelium is both odontogenic and non-odontogenic, which predisposes the jaws to the development of cystic pathology (12).

Neoplastic diseases of the oral cavity are a broader category. Neoplastic diseases are classified as odontogenic, non-odontogenic, and of salivary gland tissue origin (13). Oral tissues can develop benign or malignant neoplasms like any other body tissue, and oral cancer is of most concern to dental surgeons. The most frequent neoplasm in the mouth is oral squamous cell carcinoma (OSCC). OSCC is the fifth most frequent cancer globally with an estimated incidence of 400,000 new cases annually (14). Benign conditions such as ameloblastoma are also a concern, as they may also cause severe destruction of the jaw bone.

Oral manifestations of systemic disease may be caused by vitamin deficiencies, blood dyscrasias, metabolic disturbances, endocrine disturbances, granulomatous diseases, dermatological and mucous membrane diseases, bone diseases, and poisoning due to metals (15). Oral health providers must maintain alertness to these manifestations as the systemic disease may first present in this form. Close consideration should therefore be given to the exact pathology of oral disorders. This includes a broad spectrum of diseases ranging from modest to severe tissue damage, which will pose management challenges. Raising the awareness of oral diseases will increase the focus on the dental needs of society. To summarize, the major risk factors for oral diseases can be broadly put into three categories: dental caries, periodontal diseases, and neoplasms of the oral cavity.

## Risk Factors

### Dental Caries

Initially, the factors such as diet, microbial flora, and susceptible teeth were linked to the initiation and progression of dental caries (16). However, recent concepts state that caries is a resultant of factors such as a susceptible host, cariogenic micro-organisms, suitable substrate (sucrose sugar/carbohydrate), and duration of exposure (17). The four factors in the etiology of dental caries are influenced by local and general factors. Tooth alignment, salivary rate, and oral hygiene are local risk factors. Whereas, parameters such as gender, age, ethnicity, geographic variations, and socio-cultural practices are general risk factors for caries development. Diet is the most dominant variable and risk factor in establishing the prevalence and incidence of dental caries. The evidence suggests that microbial flora have a higher risk influence in the initiation and progression of dental caries. Studies on the localization of microbial flora related to dental caries suggested *Streptococcus mutans* as a pioneer bacteria in dental caries pathology (17). Based on the production of bacteriocins and mutacins, *Streptococcus mutans* are classified into four types, namely, I, II, III, and IV. The antigenicity and virulence of *Streptococcus mutans* are evaluated by the serotypic classification (18). Based on the chemical constituents of the cell surface in *Streptococcus mutans*, strains are classified as c, e, and f serotypes (19). *Streptococcus mutans* adhere to host tissue, i.e., tooth surface through the enzymatic action of gluocsyltransferase (GTF) enzymes. Thus, the pathogenic nature is influenced by the enzymatic factor and thus linked with the serotype of the organism (20, 21). A study that investigated the diversity, commonality, and stability of *Streptococcus mutans* genotypes associated with dental caries among children, identified the high caries risk *S. mutans* genotypes (22). Based on the studies with *Streptococcus mutans* genotypes, the suggestion is that epidemiological correlation with high caries risk in the community should be researched to identify the high-risk genotypes of *Streptococcus mutans* in the community.

### Periodontal Diseases

Periodontal disease is considered a chronic disease and frequently affects all age groups, i.e., children, adolescents, adults, and the elderly (23). The periodontal structures which support teeth are important as they hold them in their anatomical position. It is not just infection that causes periodontal morbidity but other factors have a significant role in periodontal disease initiation and progression. Risk factors have a substantial impact on the response of an individual to periodontal infection. These risk factors include age, tobacco use, smoking, alcohol consumption, brushing habits, lifestyle, genetic influences, diabetes mellitus, obesity, metabolic syndrome, osteoporosis, and vitamin deficiencies (24–26). Modification of these risk factors allows us to control periodontal diseases. Understanding risk factors and the early identification of vulnerable individuals will assist a dental surgeon in planning prevention and treatment strategies for periodontal diseases (25). Based on our understanding, some of these risk factors are independent and modifiable, such as smoking and alcohol consumption. Generating awareness about modifiable risk factors and educating the public about the other risk factors in the community may raise the importance of oral health, and the rate of tooth loss may be reduced (27). Smoking is the most well-established modifiable risk factor for periodontitis. However, evidence to support a relationship between periodontal disease and nutrition, alcohol consumption, socioeconomic status, and stress levels have not been clearly established (28).

Periodontitis has been reported to affect 11% of the global population and is listed as the sixth most frequent condition in the world (29). Globally, symptoms of periodontal disease were frequently observed in adults (30, 31). A severe form of periodontal disease, aggressive periodontitis, is noted to affect 2% of teenagers worldwide. Severe periodontitis was ranked 77th among the detailed causes of Disability Adjusted Life Years (DALYs). Severe periodontitis is mentioned as a leading cause of DALYs in 9 regions of the world, namely, Australasia, Sub-Saharan Africa East, Central, East, and Southeast Asia, and Southern, Central, Tropical, and Latin America (31). It is noteworthy to mention that data on the frequency, type, and associated risk factors of periodontal disease in the Caribbean region needs to be researched.

### Oral Cancer

Tumors of the head and neck comprise an important group of neoplastic conditions of the body. The incidence of head and neck cancers is increasing in many parts of the world (32). This increase remains high despite all the advances in modern medicine. These malignancies are more prevalent in the developing world and, unfortunately, have not received satisfactory attention as have the more prevalent cancers of the developed world, like lung, breast, and colon cancer (7).

According to the World Health Organization (WHO), the most commonly diagnosed cancers in males worldwide were those of the lung, prostrate, colorectal, stomach, bladder, and oral cavity, whereas in females, it is breast, colorectal, lung, stomach, uterus, cervix, ovary, bladder, liver, and oral cavity. The data on cancer statistics suggests that oral cancer is the sixth most common cancer among men and the tenth most common cancer in women. Oral cancers are reported to be more prevalent in developing countries of the world. However, oral cancers have not received satisfactory attention as compared with other common cancers (7, 31, 33–35).

More than 95% of the carcinomas of the oral cavity are of the squamous cell type in nature. They constitute a major health problem in developing countries, representing a leading cause of death. The survival index continues to be small (50%) as compared with the progress in diagnosis and treatment of other malignant tumors (7). The risk factors include tobacco chewing, smoking, alcohol consumption, immunosuppressed condition, and diets with low levels of vitamins A and C. Inadequate consumption of vegetables and fruits may contribute to the risk of oral cancer (23, 36, 37). In the Western world, the use of tobacco and alcohol is considered to be the greatest risk factor (38). These risk factors are independent and inter-dependent. Ogden (2000) suggested that tobacco smoking is associated with 75% of overall oral cancer cases. Further, it was mentioned that tobacco smoking individuals have a 6-fold risk of developing oral cancer when compared to non-smoking individuals. The ratio was similar with persons who drink alcohol and non-drinkers. The combination of tobacco and alcohol use poses a 15-fold risk of oral cancer development in comparison with non-users (39). While tobacco and alcohol use are traditionally the greatest risk factors, other known risk factors are betel quid chewing, areca nut, narcotics, epigenetic factors, and viral infections such as Human papilloma virus (HPV), Epstein Barr virus (EBV), and Hepatitis C virus. However, certain lesions are considered to be precursor lesions to oral cancer, and these include leukoplakia, erythroplakia, actinic cheilitis, lichen planus, sideropenic dysphagia (Plummer-Vinson syndrome), submucous fibrosis, dyskeratosis congenita, and discoid lupus erythematosus (40). Recently, these lesions are termed potentially malignant disorders (41). Cancer awareness programs should be targeted at different levels of the population. One suggestion would be to target schools, individuals with occupational risks, and persons with precursor lesions to prevent the further development of precursor lesion to cancer and to prevent the occurrence of both precancer and cancer among school children individuals who have smoking or other deleterious habits.

## Tropical Environment and Oral Disease

The term tropical denotes a climatic feature with which other aspects such as soil and vegetation are correlated. The tropical geographical location is that portion of the globe where the sun passes directly overhead. The tilt in the earth's axis extends between 23°-30° latitude north and south of the equator, and it covers 38% of the total land surface (42). The influencing factors in tropical countries are major climatic subdivisions, socio-economic status, environment, food, water, and nutrition. Oral diseases that can result from exposure to sunlight are pigmentation, actinic chelitis, squamous cell carcinoma, keratocanthoma, basal cell carcinoma, and malignant melanoma (42). Traditionally, cultural and religious rituals involving the teeth and oro-facial soft tissues also have an impact on the oral and para-oral structures such as mutilations of teeth and oral soft tissues, tooth crown, and soft tissues (43). The significance of oral diseases in tropical environments should be focused primarily on nature and society (cultural and religious rituals). To summarize, the tropical phenomenon does not restrict the scope of these inquiries solely to this zone. It can also be said that physical processes, human characteristics, national borders, and the distribution of oral diseases in tropical countries do not coincide with lines of latitude.

### Inter-relation of Oral Disease With Systemic Health

Infectious oral diseases predispose to systemic involvement and potential complications due to the hematogenous spread which can result from focal infections (44). Common inflammatory conditions of the oral tissues such as dental caries, gingivitis, and periodontitis are usually precipitated by the formation of dental plaque (45). The dilation of pulpal and periodontal vasculature due to the sequelae of inflammation provides a greater surface area that facilitates the entry of microorganisms into the bloodstream. Often, the bacteremia is transient with the highest intensity limited to the first 30 min after a triggering episode (46). On occasions, this may lead to the seeding of the microorganisms in different target organs and result in systemic infections (46). It is a well-recognized concept that oral infections, especially periodontitis may affect systemic health and contribute to systemic disease development and progression. This includes cardiovascular disease, cavernous venous thrombosis, bacterial pneumonia, diabetes mellitus, and low birth weight (47). The focus on periodontal disease is due to the fact that the periodontium can serve as a reservoir for mediators such as cytokines and interleukins which can enter the systemic circulation and induce the disease process (48–50). Based on our understanding, a large body of literature has suggested that oral infections may contribute to poor systemic health and disease development. Among the systemic developments, endocarditis has been extensively studied. A future goal in this area is to identify the epidemiological data in cases of oral infections that resulted in systemic complications.

#### Prevention of Oral Diseases

Preventive oral diseases programs should be aimed at the three conventional methods, namely, primary, secondary, and tertiary (51). The preventive programs should focus on the pre-pathogenic period prior to the onset of smoking habits among early adolescents with tobacco related health hazards and social problems. Prevention strategies should also focus on the early pathogenic period with prompt referral counseling centers, whereas tertiary prevention strategies are to focus on the prevention of complicating sequelae in the disease process (51, 52). Preventive programs should be targeted at various levels to improve oral cancer awareness. School children should be educated about oral health behavior, potential damages of oral tissues, and general health due to smoking and drinking. A plan may be proposed to the school education system about oral health awareness and the impact of compromised dental and oral health may probably motivate student learning and result in positive oral behavior (53). Films about neglected oral health and its impact on general health can be shown in an educational institution to promote oral health (54). Social stigmas related to oral conditions such as cleft lip and cleft palate should be identified and special care should be provided for these students.

#### Global Burden of Oral Diseases

The burden of oral and dental disease is high, especially in the lower socioeconomic groups and in challenged individuals in both developing and developed nations across the globe. Pathological conditions in the oral cavity such as dental decay, periodontitis, tooth loss, trauma to tooth and jaw, oropharyngeal cancers, oral mucosal lesions due to systemic manifestations, HIV related oral manifestations, and periodontal tissue damage due to diabetes are the major oral health problems worldwide (55). Poor oral hygiene and poor health have the greatest influence on the quality of life of a person (56). The varied nature of oral disease patterns across nations needs to be identified and should be used in the planning of preventive oral health care programs. Identifying the risk factors locally will help in implanting proper preventive measures for oral health.

Dental caries is still a major health problem in most industrialized countries and it affects 60–90% of school aged children (57). Worldwide, the prevalence of dental caries among adults is high as the disease affects nearly 100% of the population in the majority of countries. The data published by WHO show high Decayed, Missing, and Filled—Tooth (DMFT) values in Latin America. In several industrialized countries, older people have often had their teeth extracted due to the disease process. The proportion of edentulous adults aged 65 years is high in Albania (69%) and in the USA (26%) (7, 57). Establishing oral health awareness and the importance of teeth may increase the demand for dental treatment.

The locations characterized by high incidence rates for oral cancer (excluding the lip) are found in South and Southeast Asia (e.g., Sri Lanka, India, Pakistan, and Taiwan), parts of Western (France) and Eastern Europe (Hungary, Slovakia, and Slovenia), parts of South America (Brazil, Uruguay), the Caribbean (Puerto Rico), and in the Pacific (Papua New Guinea and Melanesia). In the Caribbean, Puerto Rico has the highest reported incidence of oral cancer (>15 per 100,000). In terms of worldwide levels, Cuba has an intermediate incidence range of cancers of the oral cavity. A Cuban study that investigated the impact of heavy cigar smoking on the population reported a smoking incidence of 7.2 per 100,000 population. The data presented was stable for over the past decade (58).

#### The Economic Impact of Oral Disease

Conventionally, dental treatment is sought by persons in higher socio-economic levels, as the costs associated with treating dental and oral diseases are high. Dental Caries is recognized to be the fourth most expensive disease to treat in industrialized countries. Dental practitioners provide their treatment with or without third party payment schemes and, in most developing countries, investment in oral health care is low (59). This makes the development of preventive oral awareness and preventive care programs mandatory if we are to reduce the prevalence of oral disease. Roby et al. (60) mentioned that industrialized countries like Israel spend 12.5%, Germany 8.6%, Sweden 8%, the USA 4.2%, and the UK and Sri Lanka 3.5% of their health funds for dental care (60). Identifying the “*partnership networking*” for oral health care is suggested as a key to reducing the economic barrier. *Partnership Networking* is aimed at bringing high-level healthcare professionals through a combination of regional and international experts to collaborate with local Ministries of Health and dentists to address health care gaps and elevate preventive oral health awareness, through campaigns, and outreach health services. Finding a partnership for oral health care in terms of prevention and oral health education should be a discussion point in dental society meetings, continuing dental education programs, and conferences.

#### Global Oral Health Inequalities

A major global problem for oral disease care is the failure to implement preventive programs and a failure to understand the social determinants of oral disease. Gaps in knowledge, the separation of oral health from general health, and inadequate evidence-based data are known to be barriers that have led to global oral health inequalities. The International Association of Dental Research (IADR) addressed these three barriers and suggested that the critical gaps in knowledge be identified as this perhaps may bring oral health concepts into the public domain (61). Developing and implementing the partnership with cognate organizations, a knowledge base that uses a standard set of reporting criteria and includes a registry of implementation trials should assist in reducing the inequalities. Emphasis should be placed on identifying the significance of social determinants of oral health. Emphasis should also be placed on the importance of integrating research on oral health inequalities with the wider goal of reducing health inequality as a whole. Emphasis should also be placed on the importance of multi, inter, and trans-disciplinary research and translational research using inter- and multi-sectorial approaches. Disease prevention strategies should be developed based on upstream prevention. Strategies should be developed that is capable of local interpretation in a way that respects cultural sensitivities and socio-economic constraints. Local, regional, and country level systems should be developed for oral health promotion and healthcare that are appropriate and recognize resource implications. The issue of oral health inequalities should be raised in wider public debates with specific emphasis on underprivileged communities (60). Reducing the barriers and proposing research driven programs. Capacity building research strategies and standardized systems for measuring oral health should raise the level of oral health awareness in society.

#### Oral Disease Scenario in the Caribbean

Bönecker et al. (61) revealed that evidence of a decrease in dental caries in Latin American and Caribbean children had been noted among 5–6 and 11- to 13-year-old children. Further, they mentioned that the decrease in dental caries was less prominent in the past few years (62). A national survey in St. Vincent and the Grenadines reported a high prevalence of calculus and bleeding, especially among older children. The proportion of children with healthy periodontium ranged from 51% among 7-year old and 12% in 15 to 19 years old (62, 63). As mentioned, Puerto Rico has the highest reported incidence of oral cancer in the Caribbean and Cuba has an intermediate incidence of oral cancers (59). A database search of Caribbean studies revealed that research was focused on the Epidemiology of cariology, periodontal disease, and hygiene or home care practices. In addition, other research areas found in the database were implantology, patient education, preventive dentistry, and dental education. The research findings from Caribbean studies are summarized in Table 1.

**Table 1:** Published research findings from the Caribbean region.

**Publication year**	**Country**	**Research hypothesis**	**Research conclusion**	**Area of research**	**Type of publication**	**References**
(2014)	Puerto Rico	Epidemiology of hypodontia in 10–14 years	The prevalence of hypodontia in Puerto Rico was 6.02%.	Epidemiology – Oral Disease	Original research	(64)
(2015)	Puerto Rico	Efficacy of CPC and essential oils mouthwashes compared to a negative control mouthwash in controlling dental plaque and gingivitis	CPC mouthwash showed a reduction of gingival index scores and gingival interproximal index scores. However, these reductions were not considered clinically significant.	Preventive dentistry - Periodontology	Original research	(65)
(2018)	Puerto Rico	To investigate frequency, severity, and risk factors associated with gingival inflammation in adult populations from Kingston (Jamaica), Santo Domingo (Dominican Republic), and San Juan (Puerto Rico).	Gingival inflammation was highly prevalent, but most study subjects presented a moderate level of gingival inflammation.	Periodontology	Original research	(27)
(2013)	Puerto Rico	Estimation of Dento-Gingival Complex dimension variation based on gingival biotype.	Dentogingival complex dimensions are different for thin, mixed, and thick gingival biotypes.	Periodontology	Original research	(66)
(2015)	Puerto Rico	Evaluate the clinical efficacy of two commercially available, fluoride-free, alcohol-free mouthwashes containing either 0.075% or 0.07% cetylpyridinium chloride (CPC) in controlling established dental plaque and gingivitis compared to a non-antibacterial control mouthwash.	Participants rinsing mouth rinse containing Cetylpyridiium chloride exhibited statistically significant reductions in all the gingivitis and plaque parameters. Whereas, in those using the non-antibacterial mouthwash, significant reductions were only observed in whole mouth and interproximal plaque scores.	Preventive dentistry - Periodontology	Original research	(67)
(2013)	Puerto Rico	Evaluate the efficacy of 0.8% arginine, potassium nitrate, and sodium fluoride mouthwashes on dentine hypersensitivity reduction.	Mouthwash containing arginine provides a significant and superior reduction in dentine hypersensitivity compared to potassium nitrate	Preventive dentistry - Restorative dentistry	Original research	(68)
(2013)	Puerto Rico	Evaluate the efficacy of three regimens integrating toothpaste, toothbrush, and mouthwash in reducing dentine hypersensitivity.	Arginine regimen provided the greatest reduction in Tactile and Air-Blast dentine hypersensitivity compared to potassium. It also provides faster dentine hypersensitivity relief than the potassium regimen.	Preventive dentistry - Restorative dentistry	Original research	(69)
(2016)	Puerto Rico	To identify the types, food sources, and patterns of carbohydrates that significantly contribute to dental caries in Puerto Rican children.	Total carbohydrates, total sugars, ≥10% kilocaloric energy from total sugars, and sucrose, fructose, and inositol intake significantly increased caries risk	Preventive dentistry - Cariology	Original research	(70)
(2010)	Puerto Rico	Comparison of the efficacy of two commercially available dentifrices for the control of supragingival plaque and gingivitis.	Dentifrice containing 0.3% triclosan, 2.0% PVM/MA copolymer, and 0.243% sodium fluoride provides a significant reduction in established supragingival plaque and gingivitis	Preventive dentistry- Periodontology	Original research	(71)
(2009)	Puerto Rico	Clinical investigation of the efficacy of a commercial mouth rinse containing 0.05% cetylpyridinium chloride in reducing dental plaque.	Mouthrinse containing 0.05% CPC provides significantly greater efficacy for reducing dental plaque 12 h after use than does a control mouth rinse without 0.05% CPC.	Preventive dentistry- Periodontology	Original Research	(72)
(2016)	Puerto Rico	To estimate caries levels of 12-year-old school Puerto Ricans in 2011, and to compare results to data obtained in 1997 to explore any possible change in caries outcomes after a government health insurance (GHI) reform was implemented.	Dental caries prevalence was high and the health disparity persists between children enrolled in public and private schools after more than a decade of the GHI implementation.	Preventive dentistry- Cariology	Original research	(73)
(2008)	Puerto Rico	To assess the efficacy of a dentifrice containing 0.3% triclosan/2.0% polyvinylmethyl ether/maleic acid (PVM/MA) copolymer/0.243% sodium fluoride in a 17% dual silica base for controlling established supragingival plaque and gingivitis	The dentifrice containing 0.3% triclosan/2.0% PVM/MA copolymer/0.243% sodium fluoride in a 17% dual silica base is efficacious for the control of established supragingival plaque and gingivitis.	Preventive dentistry- Periodontology	Original Research	(74)
(2002)	Puerto Rico	To compare the long-term caries increment associated with the use of two dentifrices: (1) a test dentifrice containing 0.836% sodium monofluorophosphate (1,100 ppm F) in a dicalcium phosphate dihydrate base plus 10% xylitol; and (2) a positive control dentifrice containing 0.836% sodium monofluorophosphate (1,100 ppm F) in a dicalcium phosphate dihydrate base.	For both DFS and DFT, the increments associated with the test dentifrice containing 10% xylitol were statistically significantly lower than those associated with the positive dentifrice without xylitol (P < 0.05), with the observed reductions in caries increment exceeding 10% in for both parameters.	Preventive dentistry- Cariology	Original Research	(75)
(2016)	Puerto Rico	To evaluate the association between vitamin D levels and periodontal disease in Puerto Rican adults.	Lower serum vitamin D levels are significantly associated with periodontitis in Puerto Rican adults.	Periodontal medicine	Original Research	(76)
(2006)	Puerto Rico	To estimate the prevalence of pit and fissure sealants on first permanent molars in 12 years old living in Puerto Rico and to further evaluate dental sealant prevalence by (1) urban/rural and public/private school status as well as (2) gender;	The prevalence of dental sealants in the first permanent molars of 12-year olds living in Puerto Rico during 1997 (4.3%) is lower than that reported in the United States (18.5%). Sealant prevalence was higher in males and students attending (urban) private schools.	Preventive dentistry - Cariology	Original Research	(77)
(2003)	Puerto Rico	To assess the prevalence of dental caries amongst 12-year-old Puerto Ricans.	The mean DMFS for 12 years old is higher than the mean DMFS of 4.2–4.7, reported for 12–17 years olds in the USA. Dental caries is a highly prevalent disease amongst 12-year-old in Puerto Rico.	Epdiemiology - Cariology	Original Research	(78)
(2018)	Puerto Rico	To estimate the prevalence of gingivitis and calculus among 12-year-old Puerto Ricans	Gingivitis prevalence is higher among 12-year-old Puerto Ricans compared to data reported for U.S. adolescents.	Epidemiology - Periodontology	Original Research	(79)
(2017)	Puerto Rico	To estimate the prevalence of gingivitis in 35- to 70-year-olds residing in San Juan, Puerto Rico, and assess the differences in gingivitis distribution between age and gender groups.	Gingivitis was observed in all participants. Men had significantly higher GI, compared to women. The prevalence of gingivitis was higher in Puerto Rico than in the US.	Epidemiology - Periodontology	Original Research	(80)
(2014)	Puerto Rico	Details the strategies for engaging Caribbean dental researchers		Public health	Review	(81)
(2015)	Trinidad and Tobago	to compare the effect of Motivational Interviewing, in contrast to traditional dental health education (DHE), on oral health knowledge, attitudes, beliefs, and behaviors among parents and caregivers of preschool children in Trinidad.	Using a Motivational Interviewing approach when delivering oral health information had a positive effect on parent/ caregiver oral health knowledge, attitudes, and behaviors compared to traditional Dental Health Education.	Preventive dentistry-Patient education	Original Research	(82)
(2013)	Trinidad and Tobago	To describe the prevalence and severity of early childhood caries in preschool children in a region of central Trinidad and to explore its relationship with social and behavioral factors	The prevalence and severity of ECC in central Trinidad were related to oral health behaviors and access to dental care.	Epidemiology - Cariology	Original Research	(83)
(2015)	Trinidad and Tobago	To describe the prevalence of missing teeth, use of bridges and dentures, and unmet dental needs among those aged 60 years and above.	The prevalence of missing teeth, use of bridges and dentures, and unmet dental needs were high in the SABE cities in 1999–2000.	Epidemiology – Geriatrics	Original Research	(84)
(2012)	Trinidad and Tobago	To explore and understand parents and caregivers' experience of oral healthcare for their preschool aged children and how, within their own social context, this may have shaped their oral health attitudes and behaviors	Parents and caregivers in this qualitative study showed generally positive attitudes toward oral health but appear to have encountered several barriers and challenges to achieving ideal preventive care for their child, with respect to a healthy diet, good oral hygiene, and dental attendance.	Preventive dentistry-Patient education	Original Research	(85)
(2016)	Trinidad and Tobago	The purpose of this study was to describe the prevalence of developmental defects of enamel (DDE) and their relationship with early childhood caries (ECC) among preschool children in Trinidad.	Developmental Defects of Enamel are prevalent among this group of preschool children in Trinidad and are risk factors for Early childhood caries, which emphasizes the importance of preventive oral health care in early childhood for these high-risk children.	Epidemiology – Oral disease	Original Research	(86)
(2016)	Trinidad and Tobago	To describe the relationship between oral health-related quality of life (OHRQoL) and Early Childhood Caries (ECC) among preschool children in a Caribbean population.	The study sample of preschool children OHRQoL was associated with ECC	Preventive dentistry	Original Research	(87)
(2002)	Trinidad and Tobago	To investigate sources of stress and psychological disturbance in dental students across the 5 years of undergraduate study at a dental school in Trinidad.	A psychological disturbance was significantly associated with stress levels for male students but not generally for female students.	Dental Education	Original Research	(88)
(2004)	Trinidad and Tobago	To describe levels of self-rated competency of dental graduates from the University of the West Indies (UWI) and to investigate relationships with gender and the effect of curriculum change	Female graduates rated four competencies significantly higher than males. Graduates exposed to the new curriculum perceived greater overall preparedness for a general dental practice, suggesting the change to a competency-based curriculum was effective.	Dental Education	Original Research	(89)
(2003)	Trinidad and Tobago	To determine the views of dental students concerning the acceptability of the use of sedation in the management of dentally anxious children.	Dental students' perceptions of the acceptability of interventions for use with dentally anxious patients are related to the effectiveness of the intervention. Sedation, regardless of the outcome, is seen as less acceptable than the use of rewards and relaxation.	Dental Education	Original Research	(90)
(2008)	Trinidad and Tobago	To describe parents' views on the dental health of pre-school children in Trinidad.	The generally inaccurate factual knowledge and low awareness of preventive care among parents suggest the need for accurate information about factors influencing the dental health of pre-school children.	Preventive Dentistry	Original Research	(91)
(2013)	Dominican Republic	To evaluate the clinical efficacy of a single professional application of a Pro-Relief desensitizing fluoride-free paste containing 8% arginine and calcium as compared to a fluoride-free prophylaxis paste on dentin hypersensitivity	Single professional application of Pro-Relief desensitizing fluoride-free paste containing 8% arginine and calcium carbonate provided a greater level of instant relief of dentin hypersensitvity that differs significantly from that of a fluoride free prophy paste.	Preventive dentistry - Restorative dentistry	Original research	(92)
(2020)	Dominican Republic	To achieve consensus on the learning domains of cariology education among undergraduate dental schools in the Caribbean countries.	The consensus was obtained from 15 participating dental schools in the Caribbean region.	Restorative dentistry and dental education	Original research	(93)
(2014)	Dominican Republic	To evaluate the healing of extraction sockets after implantation of biphasic calcium sulfate (CS) alone or in combination with a gamma-radiated human mineralized allograft.	Biphasic CS used alone or in combination with an allograft resulted in the same amount of NB formation in alveolar ridge preservation procedures.	Implantology	Original research	(94)
(2013)	Dominican Republic	To evaluate the histometric characteristics of the peri-implant mucosa of human subjects that received textured implant abutments with conventional (implant and abutment with the same diameter) or platform-switched (implant diameter wider than that of the abutment) configurations	The different configurations between the groups tested, the apical extension of the junctional epithelium, an apical extension of the inflammatory cell infiltrate, and maximum occupied by inflammatory cells did not differ between groups.	Implantology	Original research	(95)
(2005)	Dominican Republic	To estimate the prevalence of periodontal attachment loss among Dominican adolescents.	Clinical attachment loss is common in adolescents in Santo Domingo, Dominican Republic, suggesting the necessity for improved standards of prevention, diagnosis, and treatment of these lesions.	Epidemiology Periodontology	Original research	(96)
(2019)	Dominican Republic	To investigate the oral health related quality of life associated with gingival parameters on the Caribbean adult population.	The study identified modifiable risk factors associated with poor oral health related quality of life among participants from the Caribbean region.	Periodontology	Original research	(56)
(2018)	Dominican Republic	To investigate the efficacy of commercial Chlorhexidine mouth rinses available from the Dominican Republic. The efficacy was investigated focusing on antibacterial, anti-inflammatory, and matrix metalloproteinases-8 (MMP-8) action.	Commercial Chlorhexidine digluconate mouth rinses demonstrated inhibition of plaque in concentrations of 0.12 and 0.15%.	Periodontology	Original research	(10)
(2016)	Dominican Republic	To compare the periodonto-pathogen prevalence and tetracycline resistance genes in Dominican patients with different periodontal conditions.	Red complex bacteria and D. pneumosintes were significantly the most prevalent species among periodontitis patients. T. forsythia was the most frequently detected in this population	Periodontology	Original research	(97)
(2015)	Dominican Republic	To evaluate the effect of the platform-switching phenomenon, the use of a smaller diameter abutment on a larger diameter implant platform.	Histological findings for both conventional and platform-switched implant-abutment configurations showed a similar composition of the soft tissue	Implantology	Original research	(98)
(2019)	Jamaica	To investigate the frequency and compare HPV strains in HIV and non-HIV Jamaicans.	HPV prevalence was 8.65%. HPV 84 was the most common type in both HIV and non HIV patients.	Microbiology	Original research	(99)
(2016)	Jamaica	To investigate frequency and gender disparity of torus (palatinus and mandibularis) among UWI medical and dental students.	The frequency of torus was 27.76%. The prevalence rate is comparatively higher than neighboring countries in the Caribbean region.	Oral disease	Original research	(100)
(2020)	Jamaica	To investigate the frequency of smoking practice and gingival inflammation in three nations (Jamaica, Dominican Republic, and Puerto Rico) from the Caribbean region.	Smoking was most prevalent among Jamaicans and least in Dominican republicans. The study findings also showed that smokers have a 4-fold increased risk for developing severe gingival inflammation; and 2-fold increased risk for developing moderate gingival inflammation.	Periodontology	Original research	(26)

#### Nutrition and Oral Disease

Nutrition significantly influences the development and progression of dental and oral tissues (101). The nutritional impact on dental and oral diseases can result from either high sugar content or malnutrition. Dental caries is the most common condition that arises due to the nutritional status of a person. However, other factors also play a role in the initiation and development of carious teeth (2, 93). Nutrition-related pathologies that affect oral tissues are dental caries, periodontal diseases, erosions, fluorosis, acute necrotizing ulcerative gingivitis (102) or periodontitis or oral manifestations of avitaminoses, and micro- or macro-mineral deficiencies. Malnutrition also influences the development and growth of the dentition (103). Dental caries results from acids synthesized by cariogenic bacteria and carbohydrate sources. Oral manifestations in Vitamin B deficiency show glossitis due to loss of papilla over the dorsum of the tongue. Atrophy of fungiform and filliform papillae is observed in folic acid deficiency (104). Vitamin C deficiency presents with bleeding and spongy appearance of the gingiva. Vitamin A and D deficiencies may present as enamel hypoplasia. Vitamin A influences the turnover rate of keratinized cells. Thus, vitamin A deficiency may affect the exfoliation of oral epithelial cells and ulcerations. Vitamin K deficiency presents with wider pre-dentin thickness over the tooth (105). Minerals such as zinc, calcium, manganese, copper, magnesium, and selenium also show oral manifestations. Burning sensations of the tongue or oral cavity are associated with zinc deficiencies (106). Calcium deficiency during the growth or eruption of teeth may result in enamel hypoplasia (107). Other micro mineral deficiencies may show oral ulcerations and impaired wound healing. Acute necrotizing ulcerative gingivitis or periodontitis are usually observed in individuals with malnutrition (102).

#### Future Directions

Data about the global burden of oral diseases is well-documented but finding data on the oral disease status in Caribbean populations is difficult to identify among those published documents. Epidemiological research (cross sectional and longitudinal studies) on oral diseases needs to be documented on Caribbean populations. Conducting a survey with dental surgeons about their practice and experience of dental disease in the country may generate immediate documentation about the oral disease prevalence. A special focus should be made on dental caries, periodontal diseases, fluorosis, edentulouness, and oral cancer. A survey of dental surgeons that focuses on oral cancer patients in their care, similarly, ENT practitioners and medical hospitals may also assist in generating needed data on the oral cancer burden in the Caribbean population. The survey questionnaire should also include questions on habits such as smoking, alcohol, marijuana (ganja) usage, and other relevant data. The data on habits may be useful for sub-analysis of the survey questions with the disease-like risk factors. Data from cancer registries will be helpful in analyzing the risk factors for oral cancer. The need for epidemiological and surveillance studies to determine the scope of oral health problems and their impact on future dental services needs to be stressed to oral health care workers in meetings. The National Institute of Cancer in the United States of America conducts a Surveillance, Epidemiology, and End result or “SEER” program. A proposal for SEER like programs needs to be planned in the Caribbean and by developing such programs, oral cancer data will be generated in a continuous mode. Interdisciplinary studies such as oral health in HIV/AIDS, oral health in psychiatric patients, oral health in physically compromised individuals, oral mucosal lesions in patients with dermatological diseases, and periodontal health status in Type II Diabetic patients should be carried out. Studies need to be proposed for hospital-based patients such as “oral hygiene evaluation in physically and mentally challenged individuals.” Documentation of oral findings in systemic disease may strengthen the trans or multi-disciplinary approach to oral health care. Such trans or multi-disciplinary studies should be promoted in dental clinics with the medical hospital or educational institutional setups. Creating knowledge about “the importance of the primary dentition” in schoolage children between the ages of 5–13 may reverse the trend in dental diseases in the future. In school based oral health programs, information about diet and its role in dental and oral health have to be included, and the same information should be made available to parents. Conducting surveys relating to the “knowledge about oral cancer and its awareness” in individuals in the age group of 16–25 years across these educational institutions can be planned to determine the level of “awareness.” Based on the results of these survey reports, oral health care providers may understand the level of awareness, and this will be a helpful tool in revising existing oral cancer awareness programs. A number of studies have been made on tobacco smoking or chewing and oral cancer.

Jamaica has a longstanding reputation for ganja usage. Ganja is widely used for recreational, medicinal (folk medicine), and religious purposes in Jamaica. A report by the National Commission on Ganja in Jamaica suggested that one-third of ganja users started their habit at the age of 19 or below (41). Studies need to be proposed to know the “effect on the oral mucosa from smoking ganja.” The suggestion of oral mucosal evaluation in ganja smokers is being made because it is not just the carcinogens in the tobacco or any agent that is responsible for cancer formation, but the heat generated during smoking may result in a genetic assault resulting in cancer formation. Soyibo et al. mentioned that the use of drugs is relatively common among high school students in Jamaica (108). Awareness measures, such as screening camps and health talks, need to be promoted to school children at their educational institutions. The inclusion of educational courses on general and oral health in the school education system at all levels may reinforce the benefits of good oral health. Statistics on oral and maxillofacial injuries due to road traffic accidents may be helpful in creating trauma care centers.

Research needs to be funded properly in order for proper research to be carried out. Promoting research also needs funding sources, and hence, the identification of associations or institutions that can provide research funding is essential. Whereas, local associations would be ideal, many of these have depleted resources due to the impact of the local economic climate. Thus, the next area of possible funding is from regional or international associations. Finding a regional source in such associations would greatly support and enhance research activities. Globally, major dental research projects are conducted with the grant support of the International Association of Dental Research (IADR). Recently, a proposal for new regional developments in the IADR has been approved for the Caribbean region (109). IADR collaboration in the Caribbean region may assist researchers to collaborate and develop future dental research standards in this region. This is an opportunity that Caribbean researchers must take as it will provide an additional funding branch for their Research program. Thus, a directed approach to research not only increases the statistical data about dental and oral diseases in the Caribbean population but also raises awareness among the Caribbean population.

## Conclusion

Despite a large number of data available on the global burden of oral diseases, the data on the Caribbean population is less. It is important to document the prevalence of various oral diseases in the Caribbean population for making oral health care policies. The data generated may be helpful to determine the oral health care required and, thus, eventually raise the concept of awareness in oral diseases. Directing the oral health care awareness program in a specific way will reduce the burden of oral diseases in the Caribbean population.

## Author Contributions

ABRS made substantial contributions to the concepts, design, and intellectual content of the study and manuscript, involved in the preparation, editing, and review of the manuscript. TJ participated in manuscript writing concept, design, intellectual content of the study literature data acquisition, manuscript writing, and manuscript review. All authors contributed to the article and approved the submitted version.

## Conflict of Interest

The authors declare that the research was conducted in the absence of any commercial or financial relationships that could be construed as a potential conflict of interest.

## Publisher's Note

All claims expressed in this article are solely those of the authors and do not necessarily represent those of their affiliated organizations, or those of the publisher, the editors and the reviewers. Any product that may be evaluated in this article, or claim that may be made by its manufacturer, is not guaranteed or endorsed by the publisher.
